# Convergence in Sleep Time Accomplished? Gender Gap in Sleep Time for Middle-Aged Adults in Korea

**DOI:** 10.3390/ijerph15040803

**Published:** 2018-04-19

**Authors:** Seung-Eun Cha, Ki-Soo Eun

**Affiliations:** 1 Department of Child and Family Welfare, University of Suwon, 17 Wauangill, Bongdam-eup, Hwasung-si, Gyounggi-do 18323, Korea; secha@suwon.ac.kr; 2 Graduate School of International Studies, Seoul National University, Gwanak-ro, Gwanak-gu, Seoul 08826, Korea

**Keywords:** gender gap in sleep time, quality of life, sleep trend, time use, work-life balance

## Abstract

Although the gender gap in sleep time has narrowed significantly in the last decade, middle-aged women between ages 35 and 60 still sleep less than their male counterparts in Korea. This study examines and provides evidence for factors contributing to the gender gap in this age group. Using Korean Time Use Survey (KTUS) data from 2004, 2009 and 2014, we find that middle-aged women’s difficulty in managing work-life balance and traditional role expectations placed upon women are the main causes of the gender gap in sleep time. The decomposition analysis reveals that the improved socioeconomic status and recent changes in familial expectations for women may have helped them sleep more than in the past. However, there remain fundamental differences in attitude and time use patterns between men and women that prevent middle-aged women from getting the same amount of sleep.

## 1. Introduction

We live in a 24/7 society where more people control their sleep time and become virtually sleepless in some cases in order to conquer the night [1]. Recent studies on sleep argue that sleep is not only biologically constructed but also socially embedded. To investigate one’s sleep pattern can provide a window into the individual’s quality of life (QoL) [2,3]. A strong association between sleep experience and one’s well-being has prompted abundant discussions on problems resulting from sleep deprivation [4,5,6,7,8,9,10]. However, only a limited number of studies so far have investigated how sleep experience varies among different social groups and why [11,12,13,14,15,16].

Some scholars focused on examining a gender gap in sleep experiences, yet their findings have produced mixed results [16]. For instance, some scholars point out that insufficient sleep and daytime drowsiness were more prevalent among women than men [17,18]. However, others insist that men sleep less on average [19,20,21,22]. A U.K. study argues that women complain more about their sleep than men even though there was little variance in sleep hours by gender [2]. Interestingly, prior studies done in Asia point to a consistent gender difference in sleep time. Women complained more about their sleep than men and reported fewer sleep hours than men in Korea, Japan and Taiwan [23,24,25,26]. In particular, the gap was most pronounced for middle-aged women.

A report on women’s lack of sleep in Korea was first published in 2000. Based on Korean Time Use Survey (KTUS) data from 1999, it was revealed that married women tended to sleep less than any other group in Korean society [27]. In Korea, recently, we observe a slow but gradual conversion in time use patterns for paid work and/or housework between men and women [28]. The time use patterns of an employed woman, for instance, look more similar to her male counterpart’s today compared to 15 years ago [29]. Another study found that the time allocation for paid work and domestic work between men and women narrowed [30].

However, scholars have warned that gender equality in time allocation is very difficult to achieve. Empirical data show that the progress towards gender convergence in time use has been stalled recently [31]. Current studies on the Western societies report that even if men share unpaid work with their spouses, other forms of gender inequality still emerge in non-household related domains, such as time spent on leisure, exercise or rest [32,33].

Then, has sleep time also converged for both men and women over time? Recent KTUS data from 2014 reveal that Korean people sleep about 11 more min per day than they did in the past. Similar trends were found in Japan as well [26]. This overall increase in sleep time for the general population helped to narrow the gender gap. Nonetheless, women between ages 35 and 60 in particular still enjoy less sleep than men in the same age group. It led us to ask two questions: Why do women between ages 35 and 60 tend to sleep less than men? Will this gap remain in the future or eventually disappear?

The basic assumption for such gap in prior studies was that men and women spend their time differently and that the discrepancy in sleep time could diminish if the time use patterns of men and women became similar [25,34]. On the other hand, public health studies found that experiencing social discrimination produces social stress for an individual, and that such stress may interfere with one’s sleep. Arber and her colleagues argued that socioeconomic status gradients, such as the level of education or wage and income differences may also account for the discrepancy in sleep experience between men and women [2,35,36] (see more in Arber et al. [2]).

The issue of difficulties in work-life balance has been frequently raised as a contributing factor for the gender gap in sleep time. Women tend to experience more hardships seeking a balance between work and family [37,38,39]. In terms of total labor hours, summing up paid and unpaid work, married women show longer labor hours than married men [40,41]. Studies on married couples in particular illustrate that women who are both married and employed face an uphill climb when their spouses cannot help with housework and/or company (or public) policy does not provide assistance [42]. Other studies have found that the problem exacerbates with having children or an elderly person to care for [16,23].

Another issue frequently mentioned by prior studies is psychological stress. Women’s sleep can be easily interrupted if they are feeling stressed, especially at night time [43]. The ‘fourth shift’ often describes night time which is filled with more activities and responsibilities after work because tasks that occur at night are often considered a mother’s duty [44]. KTUS data from 2004 show that mothers with adolescents surprisingly sleep less than those with young children (under age 6), while child’s age did not affect fathers’ sleep [23]. Consequently, within the Korean context, we suspect that child-related stress in midlife for women could act as an important factor to explain the uneven sleeping hours for men and women.

All of these various factors were considered in conjunction with our time use survey data to identify the main drivers that contribute to the gender gap in sleep time. Such examples highlight the importance of understanding how social and psychological factors affect the gender discrepancy in sleep time in order to develop a strategy that can close the gap.

This study is driven by two research questions that are fundamental to addressing the issue. How has the gender gap in sleep time evolved over time? Is convergence occurring? What social process and psychological factors contributed to the gender gap in sleep time amongst middle-aged people during the last 10 years? By answering these questions, we aim to explore potential solutions to narrow the gap and predict how men and women’s sleep experiences may look in the future.

## 2. Data and Methods

### 2.1. Data and Measurements

We used the Korean Time Use Survey (KTUS) data for the analysis of sleep time by gender. KTUS data are nationally representative cross-sectional data collected by Statistics Korea every five years. The data are gathered from face-to-face interviews, where all members of a household over the age of 10 are asked to keep track of all of their activities for two consecutive days. The participants are asked to begin journaling at 00:00 hour and record their activities in 10-min slots until 23:50 each day. For this particular research, we used KTUS data from 2004, 2009 and 2014. The pooled data consist of 157,770 diaries with participants ranging from age 10 to 104. From this original data, we selected a sub-sample of adults between ages 35 to 60 (N = 70,678). The mean age of this analytic sub-sample was 46.16. Weekday diaries make up 60% of the total diary sample, while 24.3% of participants were unemployed.

#### 2.1.1. Main Dependent Variable

The sleep time used in this study is the sum of all the spells of time diary for sleep reported in 24 h. This definition can be applied to all three datasets of time diaries that consist of equal 10-min slots. The sleep time variable in this study includes both the extended hours of sleep and naps. We need to note that the quality of sleep was not captured in KTUS data, thus sleep time is presumed to be whenever the person was in bed in this study.

#### 2.1.2. Time Use Variables

Domestic chores include but are not limited to: cooking, baking, cleaning, doing laundry, maintaining house or car and running errands. Hours of ‘care’ are the sum of hours spent caring for young children (all activities related to physical care and nursing, reading, playing, helping with studies, etc.), adolescents, spouses and elderly family members. Overworked hours are measured by how long the respondents worked per week in their main job as well as side jobs, if any. The average weekly work hours for an employed individual were 49 h according to the KTUS data. In order to distinguish respondents by their workloads, we categorized them into two types: the ‘overworked’, who worked more than 50 h per week, and the ‘others’, who worked less than 50 h per week. Free time was calculated by subtracting the obligatory time (the sum of hours for paid work, domestic chores, care work and commute), sleep time and other personal care time (eating, drinking, health treatment, etc.) from the total time used per day (1440 min). Using the median free time of the sample, free time measures were categorized into three groups: short, medium and long.

#### 2.1.3. Other Variables included in the Model

The difference in sleep time during weekdays and weekends was pronounced in prior research. Sundays seem to be the slumber day for many Koreans as the amount of sleep is significantly higher on Sundays than others. After the implementation of the five-day work week policy in 2004 in Korea, the overall sleep hours increased in 2009 and 2014, driven by the increase in sleep time on Saturdays. The day of the week was an important control variable to detect sleep determinants in this research.

According to previous studies, child birth and menopause are associated with sleep uneasiness or short sleep among women [45]. Thus, considering health can be important when examining the gender gap in sleep hours [46]. Unfortunately, only the KTUS data from 2014 collected self-rated health conditions. This study used age as the proxy for respondents’ health conditions. We enter the age and age-square term into the equation, as the sleep trend by age is known to form a curvilinear trend for women.

Income level is measured in 11 levels of monthly wages ranging from none (1) to more than 700 million Korean won, about $5400 (11). Education level is categorized into four groups: junior high, high school, 2-year college, and 4-year university. Proportion of urban vs. rural population amongst the sample shows that most respondents are city dwellers. Marital status and employment status are included in the analysis as a dichotomous variable.

Having children is closely related with sleep deprivation for women. However, the three KTUS did not collect ages of the youngest child in a household in a same way. The only commonly available variable in all three surveys is ‘whether the household had a young child or children under the age of 10′. Hence, we categorized the households with children into two groups: those with a young child or children under the age of 10 (ages 9 and younger), and those with a child or children ages 10 and over.

Time pressure is used to indicate the level of psychological stress in this study measured by the question, “How often do you feel busy or rushed?” on a four-point Likert-type scale (1: never, 2: seldom, 3: sometimes, 4: always). One’s attitude towards gender role was measured by asking the respondents, “How strongly do you agree with the statement: ‘Men are breadwinners and housework is women’s duty’”. The response was measured on a four-point Likert-type scale (1: strongly agree, 2: agree, 3: disagree, 4: strongly disagree); the higher the rating, the more one would be considered to prefer equal gender roles. Afterwards, time pressure and gender-role attitude are grouped into two groups by recoding (0: not pressured, 1: time-pressured, and 0: traditional gender roles, 1: equal gender roles). Lastly, yearly dummies are included as important controls as sleep hours gradually increased over time. Descriptions of the sample are presented in Table 1 by each year.

### 2.2. Analytic Procedures

Before we test the factors relevant to the gender gap in sleep, we examine how men and women’s sleep hours differ by age and how it changes over the years. Next, we check the compositional difference of the factors relevant to the sleep time of men and women in order to identify the in-group difference. Then, we run a series of hierarchical regression models to identify the social determinants of the gender gap in sleep hours:

Model 1 = gender + year + day of week + age + age-square (baseline model)
Model 2 = Model 1 + education + wage + employment status (social inequality)
Model 3 = Mode 2 + marital status + hours of domestic chores + hours of care work (family role)
Model 4 = Model 3 + overwork + have child under age 10 (difficulties in work-life balance)
Model 5 = Model 4 + time pressure+ gender role attitudes (psychosocial factors)

Model 1 examines the initial gender gap in sleep hours with control dummies for the year, day of the week, and age with age square term. In Model 2, in order to test the socioeconomic status difference by gender, we add the level of education, level of income from work, and employment status in addition to variables included in Model 1. Next, Model 3 tests the association between family role related variables and the gender gap in sleep; marital status, hours of domestic chores and hours of care work on a daily basis are considered. Model 4 examines difficulties in work-life balance and the contribution of motherhood to the gender gap in sleep exacerbated with the child factor and the burden of excessive work on a weekly basis. Finally, in Model 5, we add two additional variables to test the ‘fourth shift phenomena’ (time pressure and gender role attitudes) of attitudes and psychological differences by gender. Descriptions of samples and details of the distribution of variables by gender are presented in Table 1.

Additionally, we conduct the Oaxaca-Blinder decomposition analysis to split the relevant factors into endowment and coefficient. Examining the differences in social factors for sleep may reveal the nature of the sleep time gap and tell us whether it stems from differences in socio-demographic characteristics that tend to change over time or due to sensitivity and attitude or other, unexplained factor, maybe, cultural issues related to sleep [47,48]. We use STATA version 12 (Jason TG, Seoul Korea) for the analysis.

## 3. Results

### 3.1. Gender Gap in Sleep: Changes over the Decade

Table 2 displays the gender gap in sleep duration between men and women between ages 35 and 60 for the three survey years (2004, 2009 and 2014). The gaps are statistically significant for each year even when the day of week and age (age and age-square) are considered. In 2004, women, on average, slept 15 min less than men per day. That makes 105 min less sleep for women than men each week. However, such gap in sleep was almost halved in 2009 (7 min), and in 2014 (almost 9 min). The regression analysis shows that the gap actually narrows down in the last 10 years, yet it needs to be noted that the gender gap in sleep hour is still statistically significant.

### 3.2. Compositional Difference in Sleep Hour of Men and Women

Table 3 presents the separate gender model that tests social determinants of sleep time. The variables of year, day of week and age are significantly associated with sleep. As expected, both men and women slept more in 2009 and 2014 compared to 2004, and the weekends were slumber days for both genders.

From Table 3, we notice a similar pattern for both men and women: the higher the education level, the stronger the feeling of time pressure, and the more overtime work on a weekly basis, the shorter the sleep duration for both genders. Interestingly, both men and women sleep more hours if they have a young child (under the age of 10) in the household compared to having an older child.

We find notable differences when examining the regression coefficients by gender. The relationship between sleep and age including age-square term is statistically significant only for women. Men’s sleep time decreases linearly while women’s sleep time decreases and then increases between age 35 and 60. While having a spouse and having to dedicate more hours to caretaking cause the sleep time for men to increase, the same factors curtail sleep hours for women. This contrasting role of family-related factors is also shown to be valid in the employed group model. Even the increase in the hours of domestic chores is linked to an increase in sleep duration for employed men. This implies that employed men, who need to partake in long hours of housework or caretaking for family members, are able to find time to sleep more. This may be possible due to having a part-time job or flexibility in managing their work hours. However, such beneficial associations are not found among employed women.

Another important difference is detected in the income level and gender-role attitudes. For men, higher income level is associated with shorter sleep hours, while it is not demonstrated in women. In terms of gender role attitude, men’s sleep time does not have any relation with gender role attitude whereas women’s sleep time is significantly different by gender role attitude.

**Figure 1:**
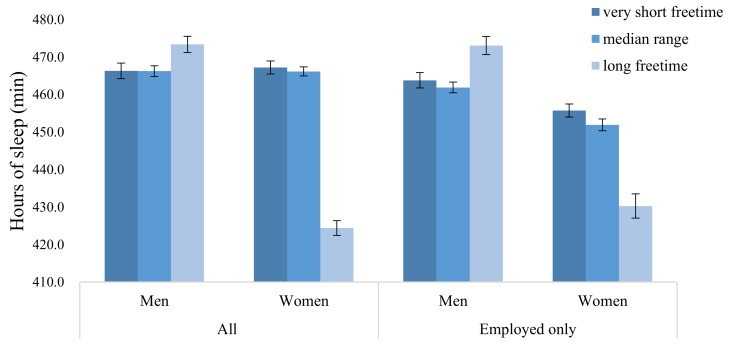
Marginal means of sleep time by gender and three free time categories: All and the employed only.

Additionally, working overtime is highly correlated with free time, thus we are not able to include free time variable with working overtime variable in the analytic model displayed in Table 3. Hence, we conduct an additional analysis by substituting the variable free time for the variable overwork in the regression model. Based on the refined regression model, we estimate the marginal means of sleep duration by each subgroup for free time (see Figure 1).

The results reveal that sleep hours for men are positively related to their free time. Men do not necessarily have to sacrifice their free time to sleep more. For women, however, there is a clear tradeoff between sleep time and free time. Women who sleep more tend to have less free time. Such a contrasting relationship by gender is more evident in the employed group model when compared to the pooled model.

### 3.3. Explanations for the Gender Gap in Sleep Time during Midlife

Table 4 presents a summary of the hierarchical regression model analysis of sleep time for middle-aged people. Here, we test how the gender gap in sleep hours narrows or widens as relevant variables enter the model. We pooled the three datasets and added a yearly dummy to the model. Because there are crucial differences in patterns of time use by employment status, we conduct a two-step analysis. First, we test gender difference of sleep time with the pooled sample. In the next step, we conduct the same procedure with the sub-sample of employed individuals only. The upper row of the table exhibits the pooled sample analysis, while the lower part presents the subgroup (employed individuals) analysis.

When we examine the upper part of the table, the gender gap in sleep is statistically significant (b = 11.08, *p* < 0.001), showing that among those aged 35 to 60, women sleep less than men by approximately 11 min on average. This gap increases, however, as we enter SES factors into Model 2. The results indicate that among men and women with the same education and income level, men sleep 21 min more than women. This means that women’s achieving higher SES status actually causes them to lose sleep rather than help them get more sleep.

In Model 3, by including the family role variables, the size of the coefficient is reduced from 21.42 in Model 2 to 17.01 (b = 17.01, *p* < 0.001). This indicates that the gender difference caused by SES factors is partly due to the family roles that men and women face in daily routines. As we move on to Model 4, the unstandardized coefficient of gender in sleep hours becomes even smaller than that observed in Model 3 (b = 15.92, *p* < 0.001). Model 4 tests whether difficulties in work–life balance can explain the gender gap in sleep time, and the results show that the hypothesis is partly supported. Finally, in Model 5, we add the fourth shift variables (time pressure and gender role attitudes) to test whether the difference in the gender role attitude or psychological aspects can explain the gender gap in sleep time. The coefficient drops from 15.92 to 13.46The patterns of changes of unstandardized coefficients of each model treating the employed group alone are similar to those we observe in pooled sample. However, some differences emerge as we see in the figures.

First, the initial gender gap in sleep hour (b_employed_ = −13.67) in Model 1 for the employed only is a little larger than that of the pooled sample (b_total_ = −11.08). This indicates that employed women sleep less than employed men, and this gap is larger when compared to that of the all middle-aged people.

Second, the size of the unstandardized coefficient is the largest in Model 4 treating the employed group, while the gap in sleep hours is the largest in Model 2 in all middle-aged people. The results illustrate that inequality in family roles and difficulties in work-life balance play a role in widening the gap in sleep hours between employed men and women. The disadvantage that employed women face in their duration of sleep is partly explained in Model 5.

Results show that the gap in sleep hours among employed men and women is partly associated with time pressure and the attitudes toward gender role in Korea. It is interesting that the gender coefficient in the last model for all and the employed group, respectively shows a bigger figure than that of the initial Model 1, even though relevant variables that have been mentioned in prior studies are controlled.

### 3.4. Decomposition Analysis

In the final analysis, we decompose the duration of sleep to examine the main factors that can account for the gender difference. Table 5 presents the results of decomposition of the gender gap in sleep. An average time gap of 11 min shown in the study is partly due to differences in the proportions of each variable by gender; we call this the endowment difference (27.76%). This means that if the level of education, income, employment status, burden of the role played in the family and other work-life balance characteristics are the same between men and women, the gender gap may be reduced significantly. As we have observed in earlier description of the sample, the gender gap in sleep time has narrowed over the last decade from 18 min in 2004 to 9 min in 2014. Such a reduction in the gender gap of sleep time in the last 10 years may be attributed to improved gender equality in Korean society.

However, results also show that the remaining 72.25% of the gender gap in sleep is due to the difference in coefficients and the interaction of characteristics and coefficients. We are able to observe the difference in the directions of coefficients, and the different impact that each relevant variable has for sleep time. The significant effect of coefficients implies that the sensitivity differences for social role between men and women greatly contribute to a gender gap in sleep hours. A greater part of the unexplained portion of gender difference comes from the interaction between endowment and coefficient displaying the uncharted factors in this model, such as cultural factors.

## 4. Discussion

Sleep was usually neglected for social scientific research in the past but has become a very significant research topic recently. Using population-based representative data, this study examines existing hypotheses of the gender gap in sleep time and provides evidence supporting possible factors that contribute to the gap in Korea, particularly among the middle-aged.

Gender inequality at the societal level and women’s traditional familial burden in Korea have somewhat diminished in the last decade. We were able to observe that sleep time gap between gender actually decreased significantly. The overall decline in gender gap in sleep time was partly driven by reduced housework hours, care work hours, overwork and childcare burden for women. It suggests that if the time use patterns of men and women continue to converge, the gender gap in sleep time may narrow further [25,27,38]. To achieve this, women need to attract men to share their unpaid workload and more public provisions for childcare and housework that are normally reserved for women. However, a significant gap in average sleep time between middle-aged men and women still remains in Korea according to the Korean Time Use Survey (KTUS) data from 2004, 2009 and 2014. The gap is even more apparent between employed middle-aged men and women.

Our findings from hierarchical regression model confirm that higher education is associated with less sleep for both men and women in Korea. While more women attain higher education and seek employment, employment rate is still lower for women than men in Korea, and employed women generally receive only a third of men’s income from the labor market. Therefore, disadvantages in the labor market for women must play a key role in sustaining the gender gap in sleep time, with supporting social discrimination hypothesis [2,11,36].

Midlife family challenges also seem to curtail men’s and women’s sleep but in a different way as prior studies have suggested [16,18]. The impact of the children’s age is noteworthy as it refutes the general notion that parents, especially mothers, with young children lose more sleep; they often wake up for caring children in the middle of the night. However, a prior study reported that mothers of adolescents (teenager and older) actually sleep less than any other child age group in Korea; mothers with young children (less than age 6) actually sleep as much as their spouses. This is, perhaps, due to middle-aged mothers with teenage children synchronizing their sleep schedule with their adolescent children’s [23]. Korean teenagers are likely to attend extracurricular activities until 10:00 p.m. or engage in nighttime self-study [49], which often lasts past midnight.

Surprisingly, we found that pro-gender equality attitudes in women do not necessarily lead to more sleep time for women. Initially, we assumed that women who are less responsive to traditional familial burdens placed on women would be able to sleep longer according to the bargaining hypothesis [50]. However, contrary to our assumption, the results show that women with strong gender equality attitudes actually sleep less than those who hold traditional gender views in which women of traditional gender role attitudes are expected to perform more of the unpaid work than women of gender equal attitudes. Interestingly, attitude towards gender role in either way is not significantly associated with sleep hours for men. A possible explanation for this trend may be that women who hold more progressive, pro-gender equality attitudes are more likely to seek and succeed in workplace and become victims of sleep deprivation.

Our analysis also reveals that women overall show a strong tendency to reduce their sleep time compared to men even when they both face similar levels of societal pressure or responsibilities. According to Burgard and Ailshire [16], men willingly sleep less favoring leisure activities or personal hobbies while women would much rather secure more sleep hours than pursue leisure activities or personal hobbies. Besides, this research, men’s sleep hours do not diminish as their free time increases. Mid-age Korean women clearly display a zero-sum relationship between sleep and free time, indicating that women tend to lose sleep if they wish to have more free time, and vice versa. This trade-off is more apparent among employed women with busy schedules. The results tell us that, in Korea, women tend to sacrifice their sleep if they want to enjoy more free time, while men enjoy both. We speculate that, for Korean women, sleep reduction may be the strategy for handling all their duties within 24 h, while men tend to secure more sleep hours.

Our results imply that the meaning and the selection process of reducing (or increasing) sleep differ by cultural background as well as gender. Still, we need more empirical evidence for why women in Korean society resort to demonstrating their role as both a mother and a wife through their sleep behavior and why such social expectation is so important to them. Another related question worthy of probing is whether other Asian cultures possess similar social processes that lead to gendered sleep or not.

While this research has revealed some factors that contribute to the persistent gender gap in sleep time for the middle-aged in Korea, there are several limitations to be mentioned. One of the issues on sleep is to identify how women suffer from sleep disturbance (e.g., frequent wake-up during the night, having trouble getting to sleep) and how it is associated with their total sleep hours. In TUS data we cannot detect sleep quality, which is an important limitation when using TUS data in sleep research. Another issue that remains unaddressed in this research is the relationship between sleep and health status. The presence of chronic disease or depression [6] could be closely associated with sleep. The KTUS data from 2004 and 2009 did not survey the health status of individuals, so we are unable to observe the association. These connections between sleep issues including gender difference in sleep time and health status call for further research. The relationship between the change in life stage and relevant health problem has been mentioned in the prior research [51,52]. Although we have controlled for the effect of age as a proxy of life-stage characteristics in our analysis, this is not enough to reveal the whole picture yet. Lastly, as results from decomposition analysis reveal, cultural factors can play a key part explaining the gender difference in sleep. Comparative research, searching for the different narratives of sleep among various societies would be helpful to identify the cultural difference in sleep phenomena in the future.

## 5. Conclusions

We are increasingly living in a 24/7 society where meeting the minimum required sleep time is becoming increasingly more difficult to achieve for everyone. Fierce competitions in school and workplace are likely to hinder people from enjoying sufficient sleep. Even so, middle-aged women in particular are sleeping less than their male counterparts even as the overall sleep time for men and women is converging.

The results from this study show that sleep is closely linked to social roles and different ways of management of time to fulfill them by gender. As societies keep changes towards accepting gender equality, the convergence in sleep time for men and women will also continue as it has been done in the last decade. However, it takes more than a slight shift in attitudes to promote a real change. Women with less traditional views may pursue higher education or career goals and obtain similar status as their male counterparts, but that has not translated into enjoying the same amount of sleep as men as we discussed earlier in this study. Without actual achievement of gender equality–equal pay, shared housework, public and private policies to support moms, etc., the gender gap in sleep time for the middle-aged men and women will persist. Scholars and public health professionals increasingly pay attention to sleep to improve one’s quality of life these days. Sleep is also a good measure of a society’s progress in achieving gender equality, which warrants more attention from social scientists. This is one of the important implications we can get from this study.

## Figures and Tables

**Table 1:** Characteristics of the sample: 2004, 2009, and 2014.

	2004		2009		2014	
	(*n* = 27,596)		(*n* = 18,412)		(*n* = 24,670)	
	Men	Women	Men	Women	Men	Women
Sleep Time (minutes)	466.76	451.23	465.71	458.48	471.63	462.71
Continuous variable						
Age	45.81	45.8	46.26	46.02	47.35	47.16
Domestic work (minutes)	33.53	223.37	36.72	210.89	42.08	204.93
Care work (minutes)	15.52	44.16	18.37	54.04	18.04	47.02
Income level (range: 1–11)	4.16	1.26	6.11	2.80	7.31	3.61
Categorical variable (%)						
Day of week						
weekdays	60.21	60.20	59.74	59.98	59.89	60.07
Saturday	19.84	19.95	20.10	20.03	19.85	19.79
Sunday	19.94	19.85	20.16	19.99	20.26	20.15
Education						
middle school and less	24.14	41.80	17.36	28.99	12.31	18.90
high school	44.90	43.20	45.35	48.38	41.25	46.41
some college	5.62	3.87	11.52	9.08	14.35	14.33
above university	35.34	11.13	25.78	13.54	32.08	20.36
Urban/rural						
rural	9.06	9.48	5.51	5.82	5.72	5.60
urban	90.94	90.52	94.49	94.18	94.28	94.4
Marital status						
single	5.03	1.75	6.18	2.48	8.58	4.18
with spouse	94.97	98.25	93.82	97.52	91.42	95.82
Employment status						
not employed	9.76	38.82	8.73	39.68	7.60	37.91
employed	90.24	61.18	91.27	60.32	92.40	62.09
Those who overworked	41.40	24.21	40.62	20.42	33.84	15.30
Those who have child less than age 10	29.97	22.32	25.53	19.70	26.66	21.63
Those who feel time pressed	83.01	64.10	77.35	73.68	68.17	62.76
Those who have gender equal attitudes	44.72	60.26	49.78	63.62	52.60	71.01
Those who have few free time	21.71	24.88	23.47	26.90	28.56	28.24

Note: We tested the difference of sleep time, domestic work time and care work time between middle aged men and women. The differences between men and women were statistically significant at 0.01 level without controls. More robust statistical significance test of the gender difference in sleep time with controls is shown in Table 2.

**Table 2:** Gender gap in sleep time among men and women ages 35–60: 2004, 2009, 2014.

	2004			2009			2014		
Adjusted	Coef.	(Std. Err.)		Coef.	(Std. Err.)		Coef.	(Std. Err.)	
Women	−15.56	(1.10)	***	−7.42	(1.27)	***	−8.89	(1.12)	***
Day of Week									
Saturday	15.35	(1.42)	***	27.50	(1.63)	***	34.51	(1.45)	***
Sunday	59.22	(1.43)	***	63.65	(1.63)	***	67.68	(1.44)	***
Age	−7.83	(1.06)	***	−15.55	(1.22)	***	−11.65	(1.11)	***
Age square	0.08	(0.01)	***	0.16	(0.01)	***	0.11	(0.01)	***
Constant	270.35	(25.00)	***	93.00	(28.00)	***	203.47	(25.48)	***
Number of obs.		27596			18412			24670	
F		395.72	***		355.45	***		525.44	***
R-squared		0.07			0.09			0.10	

Note: Reference group of categorical variables for gender and day of week is men and weekday. *** *p* < 0.001.

**Table 3:** Determinants of sleep time by gender: All and the employed only.

	All		The Employed only	
	Men	Women	Men	Women
	coef	coef	coef	coef
	(robust S.E.)	(robust S.E.)	(robust S.E.)	(robust S.E.)
Year (ref. year 2004)				
year 2009	3.05 *	10.00 ***	2.70	8.13 ***
	(1.54)	(1.36)	(1.59)	(1.74)
year 2014	10.91 ***	14.39 ***	10.26^***^	14.22 ***
	(1.63)	(1.41)	(1.67)	(1.78)
Week of day (ref. Weekdays)				
Saturday	27.70 ***	21.90 ***	28.57 ***	21.11 ***
	(1.33)	(1.13)	(1.41)	(1.46)
Sunday	68.46 ***	56.92 ***	71.51 ***	62.38 ***
	(1.53)	(1.26)	(1.63)	(1.67)
Age	−3.219 **	−11.63 ***	−3.386 **	−11.50 ***
	(1.22)	(1.12)	(1.27)	(1.44)
Age-square	0.023	0.115 ***	0.0247	0.113 ***
	(0.01)	(0.01)	(0.01)	(0.02)
Education	−3.82 ***	−7.10 ***	−3.11 ***	−5.22 ***
	(0.64)	(0.63)	(0.67)	(0.86)
Income level	−1.17 ***	−0.039	−1.33 ***	−0.42
	(0.28)	(0.29)	(0.29)	(0.35)
Employed	−1.65	54.35	-	-
	(14.97)	(39.44)	-	-
Married	9.28 **	−10.04 **	12.13 ***	−11.35 **
	(2.83)	(3.71)	(3.08)	(4.23)
Domestic chores (minutes)	0.03 ***	−0.04 ***	0.05 ***	−0.01
	(0.01)	(0.00)	(0.01)	(0.01)
Hours of care work (minutes)	0.01	−0.10 ***	0.01	−0.09 ***
	(0.01)	(0.01)	(0.01)	(0.01)
Presence of young child under age 10	4.01 *	19.63 ***	3.69 *	15.49 ***
	(1.70)	(1.66)	(1.74)	(2.15)
Daily Work hour(ref. no work)				
Short to normal range (else category)	−21.62	−68.08	−24.13	−68.45
	(14.82)	(39.46)	(16.17)	(40.66)
Overwork	−35.13 *	−81.65 *	−37.41 *	−79.55
	(14.83)	(39.48)	(16.18)	(40.69)
Feeling rushed	−6.04 ***	−7.37 ***	−5.63 ***	−6.99 ***
	(0.79)	(0.69)	(0.83)	(0.89)
Gender role attitude	−1.31	−2.17 **	−1.414	−2.96 **
	(0.83)	(0.76)	(0.86)	(0.96)
Constant	446.50 ***	255.40 ***	438.50 ***	306.30 ***
	(28.47)	(26.40)	(34.14)	(53.56)
R-squared	0.11 ***	0.11 ***	0.11 ***	0.11 ***
Number of obs.	34,120	36,558	31,138	22,402
(cluster num.)	(17,059)	(18,279)	(15,568)	(11,201)

* *p* < 0.05, ** *p* < 0.01, *** *p* < 0.001.

**Table 4:** Summary of Hierarchical Regression Model for Gender Gap in Sleep Time: All years (Unit: Minutes).

**All, Age 35–60 (*n* = 70,687)**															
	Model 1			Model 2			Model 3			Model 4			Model 5		
sleep	Coef.	Std. Err.		Coef.	(Std. Err.)		Coef.	Std. Err.		Coef.	Std. Err.		Coef.	(Std. Err.)	
Gender	**−11.08**	(0.67)	***	**−21.42**	(0.81)	***	**−17.01 **	(0.94)	***	**−15.92**	(0.95)	***	**−13.46 **	(0.97)	***
**The Employed Only, Age 35–60, (*n* = 53,540)**															
	Model 1			Model 2			Model 3			Model 4			Model 5		
sleep	Coef.	Std. Err.		Coef.	(Std. Err.)		Coef.	(Std. Err.)		Coef.	(Std. Err.)	P>t	Coef.	(Std. Err.)	
Gender	**−13.67**	(0.78)	***	**−18.25**	(0.91)	***	**−20.78 **	(1.06)	***	**−19.82 **	(1.08)	***	**−17.28 **	(1.11)	***

*** *p* < 0.001.

**Table 5:** Decomposition analysis on sleep time difference by gender: All and the employed only.

	All				The Employed Only			
Hours of Sleep	Coef.	(Std. Err.)	%		Coef.	(Std. Err.)	%	
Differential								
Prediction_1	468.18	(0.53)			464.82	(0.55)		
Prediction_2	457.14	(0.46)			450.86	(0.58)		
Difference	11.04	(0.70)	100.00	***	13.96	(0.80)	100.00	***
Decomposition								
Endowments	3.07	(0.90)	27.76	***	1.36	(1.07)	9.68	
Coefficients	31.46	(1.62)	284.77	***	26.60	(1.56)	190.61	***
Interaction	−23.48	(1.72)	−212.51	***	−14.00	(1.72)	−100.29	***

*** *p* < 0.001.
